# Evolving Clinical Management of Postoperative Delirium: A Narrative Review

**DOI:** 10.7759/cureus.92927

**Published:** 2025-09-22

**Authors:** Shahab Ahmadzadeh, Drake P Duplechin, Andrew T Haynes, Alex V Hollander, Ross Rieger, Carlo Jean Baptiste, Giustino Varrassi, Sahar Shekoohi, Alan D Kaye

**Affiliations:** 1 Department of Anesthesiology, Louisiana State University Health Sciences Center, Shreveport, USA; 2 School of Medicine, Louisiana State University Health Sciences Center, Shreveport, USA; 3 Pain Medicine, Fondazione Paolo Procacci, Rome, ITA

**Keywords:** anesthesia, delirium, elderly, icu, multimodal management, polypharmacy, postoperative cognitive dysfunction

## Abstract

Postoperative delirium (POD) is the most common neurologic complication after surgery, affecting 15-50% of adults ≥65 years and up to 80% of those admitted to intensive care units. Clinically, POD is characterized by an acute, fluctuating disturbance in attention, awareness, and cognition that typically arises within 72 hours of anesthesia. Its occurrence is associated with a threefold rise in 30-day mortality, prolonged mechanical ventilation, increased institutionalization, and long-term cognitive decline, underscoring its substantial human and financial burden. POD arises through a multifactorial pathogenesis that includes neuroinflammation, blood-brain‑barrier disruption, neurotransmitter imbalance, sleep fragmentation, and perioperative cerebral hypoperfusion. Patient-related risk factors (advanced age, pre-existing cognitive impairment, frailty, sensory deficits, alcohol misuse, and polypharmacy) intersect with surgical drivers such as operative duration, intraoperative hypotension, high opioid doses, benzodiazepine exposure, and postoperative infections. Prevention hinges on identifying at-risk patients and mitigating modifiable factors. High-quality evidence supports the use of multicomponent nonpharmacological bundles, including orientation protocols, early mobilization, sleep promotion, optimized pain control, and hearing and vision aids, which reduce delirium incidence by 30-40%. Among pharmacologic strategies, dexmedetomidine infusion during and after cardiac and major noncardiac surgery consistently lowers delirium rates, whereas routine prophylaxis with antipsychotics, melatonin agonists, or cholinesterase inhibitors remains unproven and is not currently recommended outside clinical trials. Management prioritizes rapid diagnosis using validated tools (e.g., confusion assessment method for the intensive care unit (CAM‑ICU)), correction of precipitating insults, and judicious symptom control with low-dose haloperidol or atypical antipsychotics when nonpharmacologic measures fail. Future research should clarify optimal hemodynamic targets, refine electroencephalogram (EEG)-guided anesthetic titration, evaluate peri‑operative neuro‑inflammation biomarkers, and develop personalized, risk-stratified prevention algorithms further to reduce POD's pervasive impact on older surgical patients.

## Introduction and background

Postoperative delirium (POD) is an acute, fluctuating disturbance of attention, awareness, and cognition that develops within hours to days after surgery and cannot be explained by a pre‑existing or evolving neurocognitive disorder [1]. Modern diagnostic frameworks include the Diagnostic and Statistical Manual of Mental Disorders, Fifth Edition (DSM‑5), and the Confusion Assessment Method (CAM); both require an abrupt change in mental status plus inattention, with either disorganized thinking or an altered level of consciousness [1]. The reported incidence ranges from 10% to 50% in general surgical cohorts and exceeds 60% after hip fracture repair or cardiac surgery, making POD the most common postoperative neurologic complication in older adults [2]. Its clinical relevance is underscored by associations with prolonged hospitalization, higher institutionalization rates, long‑term cognitive decline, and a twofold increase in one‑year mortality [3].

The pathophysiology of POD is multifactorial, reflecting a dynamic interaction between patient vulnerability, such as advanced age, dementia, frailty, sensory impairment, and polypharmacy, and perioperative insults that include anesthetic exposure, inflammation, pain, sleep disruption, and metabolic derangements [4]. Neuroinflammatory cascades, blood-brain barrier dysfunction, oxidative stress, and neurotransmitter imbalance (particularly cholinergic deficiency and dopaminergic excess) have all been implicated; however, definitive mechanistic pathways remain elusive [5]. Preventive strategies, therefore, emphasize comprehensive risk stratification and reduction of modifiable precipitants. Multicomponent, non-pharmacological bundles, orientation protocols, early mobilization, visual and hearing aids, sleep hygiene, and avoidance of deliriogenic drugs reduce the incidence of delirium by up to 40% in surgical wards [6]. Pharmacologic prophylaxis with dexmedetomidine or perioperative antipsychotics has yielded mixed results and is not universally recommended outside high‑risk settings [7]. Once delirium develops, first-line management focuses on identifying reversible medical triggers, optimizing analgesia, and providing a low-stimulation environment; pharmacological agents are reserved for severe agitation that threatens patient or staff safety [8].

Despite decades of research, real‑world implementation remains inconsistent. Screening tools, such as CAM or the 4-A's Test (4AT), are variably applied, and treatment paradigms differ across institutions, limiting the comparability of outcome data [9]. Moreover, clinical trials often exclude patients with pre‑existing cognitive impairment, a group that bears the greatest risk. Consequently, there is a pressing need for standardization of assessment methods, validation of pragmatic prevention bundles in diverse surgical populations, and mechanistic studies that can inform targeted therapies [10]. The economic imperative is equally clear: delirium adds an estimated USD 32 billion in annual healthcare costs in the United States alone, primarily through more extended hospital stays and post‑acute care utilization [11].

Given the aging surgical population and persistent practice variability, a concise synthesis of current evidence is warranted to guide perioperative teams. In this review, we will discuss the epidemiology and pathophysiology of POD, contemporary prevention strategies, and best-practice approaches to the diagnosis and management of established delirium.

## Review

Methods

To guide this narrative review, we conducted a focused literature search using the PubMed database. Articles were identified using the following search terms: “postoperative delirium,” “surgical delirium,” “risk factors,” “prevention,” and “management.” We included English-language articles published between 2010 and 2024, with an emphasis on high-quality review articles, clinical trials, and relevant observational studies. Priority was given to studies examining adult surgical populations, especially those evaluating delirium prevention strategies or validated risk assessment tools. Articles were selected based on relevance to perioperative practice, consistency with current clinical guidelines, and impact on patient outcomes.

Etiology, Risk Factors, and Pathophysiology

POD is an acute and fluctuating disturbance in attention, awareness, and cognition that commonly affects older adults following surgical procedures. It typically arises within hours to days postoperatively and is associated with poor outcomes, including prolonged hospitalization, functional decline, and increased mortality. The pathogenesis of POD is multifactorial, involving patient vulnerabilities, pharmacologic influences, and surgical stressors that converge on brain physiology. Understanding these mechanisms is essential for early identification and prevention in patient care.

Age and Preexisting Cognitive Impairment

Advanced age is a well-established, non-modifiable risk factor for POD, with incidence increasing sharply beyond age 65 [12]. Age-related vulnerabilities, such as reduced cerebral reserve, slower neurotransmitter turnover, and increased blood-brain barrier permeability, impair resilience to surgical stress. In parallel, preexisting cognitive impairment, including mild cognitive impairment and dementia, significantly elevates POD risk; over 50% of patients with dementia may develop delirium postoperatively, often with hypoactive symptoms that are easily missed [13]. Cognitive assessments, such as the Mini-Cog or the MoCA, are rarely part of routine preoperative evaluations, yet they can help identify high-risk patients. Even subjective memory complaints warrant attention, particularly when corroborated by caregivers. Incorporating cognitive screening into standard surgical planning, alongside frailty and fall assessments, may improve delirium prevention and postoperative care planning.

Alcohol Withdrawal as a Distinct Risk Factor

POD can arise from a broad array of predisposing factors, among which alcohol withdrawal remains a particularly high-risk and underrecognized contributor. Patients with a history of chronic alcohol use may develop withdrawal delirium, commonly referred to as delirium tremens, typically within 48 to 96 hours of cessation. This form of delirium is marked by autonomic instability, agitation, hallucinations, and fluctuating consciousness, and carries a high risk of morbidity and mortality if not promptly identified and managed. Given its distinct pathophysiology and treatment considerations, alcohol withdrawal delirium warrants specific attention in the perioperative setting, including early screening, prophylactic benzodiazepine use, and multidisciplinary coordination of care.

Polypharmacy and Medication Effects

Polypharmacy, typically defined as the use of five or more medications, is a major modifiable risk factor for POD, particularly in older adults. Beyond quantity, the interaction of drugs with anticholinergic, sedative, or dopaminergic properties heightens vulnerability. Common culprits include benzodiazepines, corticosteroids, opioids, and anticholinergics like diphenhydramine [13,14]. Benzodiazepines and opioids, in particular, impair cognition through GABAergic and respiratory depressant effects, especially when combined with hypoxia or sleep disruption. Many patients enter surgery already on complex medication regimens, making thoughtful perioperative medication reconciliation essential. Tools like the Beers Criteria and Anticholinergic Cognitive Burden scale can guide this process. Importantly, not only the continuation but also the abrupt withdrawal of medications (e.g., selective serotonin reuptake Inhibitors (SSRIs), beta-blockers) can precipitate delirium. Prehabilitation strategies that reduce unnecessary medications preoperatively may decrease POD risk and support better outcomes.

Pain and Sensory Impairment

Poorly controlled pain is both a physiologic and psychological stressor that heightens the risk of delirium. Pain activates the HPA axis and sympathetic system, promoting inflammation and catecholamine surges that impair cognition. Opioids, while often necessary, contribute to delirium risk through sedative and hypoxemic effects [14]. Regional anesthesia techniques have been shown to reduce both opioid use and POD incidence. In addition, sensory deficits, especially uncorrected hearing or vision loss, limit orientation and increase misinterpretation of the environment, compounding cognitive vulnerability. Simple measures such as ensuring access to hearing aids and glasses can significantly reduce delirium rates [12]. Together, effective pain control and sensory optimization should be core components of delirium prevention.

Surgical and Anesthetic Contributors

Surgical type and anesthetic technique both influence the risk of delirium through inflammatory, metabolic, and neurologic pathways. High-risk procedures such as cardiac, orthopedic, and abdominal surgeries are associated with longer durations, greater physiological stress, and increased opioid use- all contributing to higher POD rates [12,15]. Cardiac surgery with bypass, for instance, may induce cerebral microembolism and hypoperfusion, while orthopedic procedures often necessitate deep sedation and strong analgesia. Anesthetic choice also plays a role. Total intravenous anesthesia with propofol has shown some benefit in reducing POD complications compared to volatile agents, potentially due to reduced oxidative and inflammatory effects; however, the evidence remains mixed [16]. Intraoperative hypotension and deep anesthesia, especially when unmonitored, are independent delirium risk factors. EEG-guided depth monitoring may help minimize over-sedation and improve outcomes. Ultimately, anesthesiologists play a key preventive role by tailoring anesthetic plans based on a patient's cognitive and surgical risk profiles and avoiding excessive pharmacologic burden intraoperatively.

Neurotransmitter Imbalance

Neurotransmitter disruption, particularly acetylcholine deficiency, is a central mechanism in POD, impairing attention and memory. Anticholinergic medications and age-related cholinergic decline amplify this vulnerability [12]. Concurrently, dopaminergic excess is linked to agitation and hallucinations seen in hyperactive delirium, while Gamma-aminobutyric acid (GABA) and glutamate imbalances may explain hypoactive presentations and cognitive suppression [17]. Serotonin dysregulation may also contribute to sleep-wake disturbances. These complex interactions highlight why POD presents with varied phenotypes. Agents like dexmedetomidine, which modulate norepinephrine without suppressing GABA, offer targeted neurochemical stabilization. Understanding this balance supports minimizing high-risk medications and tailoring perioperative care in vulnerable patients.

Inflammatory Mechanisms

Neuroinflammation is increasingly recognized as a core pathophysiologic driver of POD. Surgical trauma induces a systemic release of pro-inflammatory cytokines, such as IL-6, IL-1β, and TNF-α, which can cross the blood-brain barrier and activate microglial cells. This glial activation disrupts synaptic function, impairs neurotransmitter balance (particularly acetylcholine and dopamine), and contributes to the acute cognitive changes seen in delirium [17]. Older adults are especially vulnerable due to "inflammaging," a state of chronic low-grade inflammation that primes the brain for exaggerated responses to surgical stress. Elevated postoperative cytokine levels, such as IL-6, have been associated with a higher risk of delirium, highlighting a potential target for preventive strategies [18]. Multimodal approaches that limit inflammation through regional anesthesia, dexmedetomidine use, or early mobilization may help attenuate this pathway.

Silent Stroke as a Diagnostic Consideration

Delirium may occasionally be the sole presenting symptom of acute neurologic events such as stroke, particularly in cases without overt motor deficits. These “silent strokes” can be easily overlooked in the postoperative setting, where delirium is often attributed to more common etiologies. Clinicians should maintain a high index of suspicion, especially in patients with risk factors such as atrial fibrillation or recent carotid interventions. In cases of sudden cognitive decline, neuroimaging, most notably a non-contrast head CT, should be considered to rule out acute cerebrovascular events.

Delirium Versus Postoperative Cognitive Dysfunction (POCD)

Although often conflated, delirium and POCD are distinct yet possibly interconnected conditions. Delirium presents acutely within days postoperatively, marked by inattention and fluctuating consciousness, and usually resolves within a week [13,15]. POCD, however, involves subtle declines in memory and executive function that can persist for months and often require formal cognitive testing to detect. While both share risk factors like age and inflammation, delirium stems from acute neurotransmitter disruption, whereas POCD may reflect longer-term neuronal injury or neurodegenerative unmasking. Notably, patients with POD have a higher risk of developing POCD, suggesting these may lie along a continuum of postoperative brain dysfunction [14].

Risk Scoring Systems

Several tools help predict POD and guide early intervention. The Confusion Assessment Method (CAM) remains the most widely used diagnostic tool due to its reliability in various clinical settings. For risk prediction, the PRE-DELIRIC model incorporates preoperative and intraoperative variables, such as age, APACHE-II score, infection, and urgency of surgery, to estimate individual risk, with strong validation in ICU cohorts [19]. The Delirium Risk Assessment Tool (DRAT) similarly considers baseline cognitive status, sensory deficits, and high-risk medications. Despite their potential, these tools are underutilized due to workflow barriers and limited EMR integration. Emerging strategies include dynamic risk models that incorporate intraoperative variables, such as anesthetic depth, and real-time monitoring. Customizing tools to specific surgical populations (e.g., cardiac vs. orthopedic) could improve accuracy. As delirium prevention becomes a surgical quality benchmark, integrating standardized risk stratification into routine pre-op evaluations is increasingly critical.

POD arises from a confluence of age-related cerebral vulnerability, pharmacologic factors, inflammatory pathways, and perioperative physiological stress. It remains a preventable and treatable syndrome with significant implications. Recognizing high-risk individuals through validated risk scores and understanding the neurobiology behind POD may enable tailored interventions, ultimately improving outcomes for surgical patients, particularly the elderly.

Diagnosis and clinical features

POD is an acute neurocognitive disorder marked by fluctuating attention, disorganized thinking, and altered consciousness, typically arising within days after surgery. According to the Diagnostic and Statistical Manual of Mental Disorders, Fifth Edition (DSM-5), delirium is diagnosed based on (A) a disturbance in attention and awareness; (B) an acute onset with a fluctuating course; (C) additional cognitive disturbances; (D) absence of better explanation by a preexisting neurocognitive disorder; and (E) a direct physiological cause such as surgery, medication, or illness [20]. Similarly, the International Classification of Diseases, Tenth Revision (ICD-10), classifies delirium under F05, defined as “delirium due to known physiological condition,” encompassing acute global brain dysfunction due to systemic factors [21].

POD manifests in three recognized clinical subtypes: hyperactive, hypoactive, and mixed. Hyperactive delirium features agitation, restlessness, mood lability, and hallucinations. Hypoactive delirium, which is more common but underrecognized, presents with lethargy, decreased responsiveness, and psychomotor retardation. Mixed delirium fluctuates between these two states. Studies suggest that hypoactive and mixed forms are frequently overlooked despite being associated with worse outcomes [12]. Notably, patients with hypoactive delirium may be mistakenly labeled as fatigued, sedated, or depressed, leading to dangerous delays in recognition and intervention.

Because of its variable presentation, several validated tools have been developed to aid in early and accurate detection. The CAM is the most widely used diagnostic tool for delirium, particularly in surgical and intensive care unit (ICU) settings. CAM diagnoses delirium based on the presence of (1) acute onset and fluctuating course, (2) inattention, and either (3) disorganized thinking or (4) altered level of consciousness [13]. CAM has demonstrated high sensitivity (94-100%) and specificity (90-95%) across clinical settings. For time-constrained environments, the 3D-CAM offers a rapid, 3-minute assessment with comparable accuracy and has been validated for use in older postoperative patients [2,22]. In some settings, it can be completed by trained nurses or non-physician providers, expanding its utility in high-volume wards.

Nurses play a pivotal role in delirium surveillance, and tools like the Nursing Delirium Screening Scale (Nu-DESC) support bedside detection. Nu-DESC evaluates five features: disorientation, inappropriate behavior, inappropriate communication, hallucinations, and psychomotor slowing, and yields a score out of 10, with ≥2 indicating possible delirium. While practical and user-friendly, Nu-DESC is less sensitive to hypoactive presentations, highlighting the need for multifaceted screening protocols [23]. Repeated Nu-DESC screening every shift, rather than once daily, can improve diagnostic yield, particularly when baseline behavior is documented and monitored over time.

Despite these tools, delirium often remains underdiagnosed. Reasons include overlap with dementia, lack of baseline cognitive assessments, staff unfamiliarity with screening tools, and the subtlety of hypoactive forms. One observational study found that over 60% of delirium cases were missed by routine care providers, emphasizing the critical need for standardized screening protocols and clinician education [12]. Delirium’s fluctuating nature further complicates detection; patients may appear lucid during morning rounds but disoriented later in the day, underscoring the need for continuous assessment and multidisciplinary communication.

Recognition and diagnosis of POD require interprofessional collaboration. Anesthesiologists can help reduce risk by optimizing intraoperative sedation and avoiding deliriogenic drugs; surgeons can anticipate risk based on surgical stress; nurses can monitor cognitive changes at the bedside; pharmacists can review medication profiles for anticholinergic or psychoactive agents; and geriatricians can aid in distinguishing delirium from dementia. Team-based care enables earlier identification, targeted prevention, and more consistent management, ultimately improving patient outcomes [12,13]. In many institutions, implementing a delirium pathway or bundle, with cognitive screening, risk stratification, and postoperative reorientation protocols, has resulted in measurable reductions in POD incidence and related complications.

Thus, POD is a dynamic and multifactorial condition that demands proactive, structured, and interdisciplinary approaches to diagnosis. While tools like the CAM, 3D-CAM, and Nu-DESC offer reliable screening methods, their effectiveness hinges on routine use, proper training, and integration into clinical workflows. Reliance on necessary recognition, particularly for hypoactive or fluctuating cases, contributes to the persistently high rates of underdiagnosis and delayed treatment. More broadly, delirium should not be seen merely as a transient, incidental occurrence in the perioperative period. Instead, its presence may signal a broader vulnerability of the brain to stress, serving as an early warning for long-term neurocognitive decline. Several longitudinal studies suggest that patients who experience POD are at increased risk for future dementia and institutionalization, reinforcing the need for delirium prevention as a pillar of surgical safety, especially in older adults [12,13]. Embedding routine cognitive screening into preoperative clinics, leveraging EMR alerts, and empowering bedside staff to act on early behavioral changes can bridge gaps in recognition and accelerate intervention. Looking ahead, innovations in digital health, such as passive sensor monitoring, speech pattern analysis, and AI-driven risk modeling, may facilitate the early detection of cognitive changes that are not visible to the human eye. Until then, vigilance, standardized assessment, and coordinated team-based care remain our best tools in the timely diagnosis and mitigation of POD.

The CAM is a widely used and validated tool for identifying delirium in clinical settings. It is based on four key features: (1) acute onset and fluctuating course, (2) inattention, (3) disorganized thinking, and (4) altered level of consciousness. A diagnosis of delirium via CAM requires the presence of features 1 and 2, plus either 3 or 4. The tool is designed to be quick and simple for non-psychiatric clinicians to administer, typically taking less than five minutes to complete. CAM’s adaptability to various settings, including general wards and postoperative care environments, has made it a standard in delirium detection protocols.

While tools like AWOL, PRE-DELIRIC, and NSQIP offer valuable frameworks for assessing delirium risk, each carries distinct limitations. The AWOL score is simple and quick to implement, but lacks external validation across diverse surgical populations. PRE-DELIRIC, although developed specifically for ICU settings with promising discrimination (AUC ~0.77-0.84), requires multiple preoperative and early ICU variables, which may not be routinely collected. NSQIP risk calculators offer general perioperative risk assessments but are not delirium-specific and may underperform in older or cognitively impaired patients. Comparative studies remain limited, and no single model has demonstrated consistent superiority. Future work should aim to validate these tools across varied surgical contexts and explore integration with electronic health records for real-time risk stratification.

While much of the existing literature focuses on ICU-related delirium, it is important to recognize that POD is also prevalent in general medical and surgical wards. Risk profiles, detection methods, and management strategies may differ significantly in non-ICU environments. Tools such as the 4AT and Nu-DESC are more commonly applied outside the ICU, and non-ICU delirium often receives less consistent attention, despite contributing substantially to morbidity, discharge delays, and institutionalization risk.

Recent advances in machine learning (ML) have shown promise in improving POD risk prediction by capturing complex, nonlinear relationships among risk factors. Models incorporating gradient boosting, random forests, and deep learning algorithms have demonstrated superior predictive accuracy compared to traditional scoring systems in select cohorts. For instance, ML-based models trained on large electronic health record datasets have achieved AUC values exceeding 0.85 in some studies. However, these models often suffer from limited external validation, a lack of interpretability, and challenges with clinical integration. Their emergence represents a key frontier in POD research, but further work is needed to ensure generalizability and transparency before widespread adoption.

Prevention strategies

Preventing POD necessitates a comprehensive, multifactorial approach involving preoperative risk stratification, medication optimization, implementation of Enhanced Recovery After Surgery (ERAS) protocols, sleep management, early mobilization, and family involvement. A well-coordinated effort across these domains is essential to reduce the incidence and severity of delirium, ultimately improving patient outcomes [24].

Preoperative screening is pivotal in identifying patients at high risk for developing delirium. Utilizing validated tools, such as the CAM and the Delirium Risk Assessment Scale, allows clinicians to assess key risk factors such as age, baseline cognitive function, and the presence of comorbidities (e.g., diabetes, cardiovascular disease) [25]. Early identification of at-risk patients enables the tailoring of perioperative management strategies designed to minimize the risk of delirium [26]. 

An essential aspect of preoperative management is a thorough medication review. Several pharmacologic agents, including benzodiazepines, anticholinergics, and opioids, are associated with an increased risk of delirium. Discontinuing or substituting these medications with safer alternatives, when possible, is a critical step in minimizing POD. Furthermore, utilizing short-acting anesthetics and avoiding polypharmacy can reduce the pharmacologic burden on patients, particularly those with multiple comorbidities, thereby reducing the risk of cognitive disturbances postoperatively [27].

ERAS protocols have been shown to reduce the incidence of postoperative complications, including delirium, significantly. These evidence-based protocols aim to optimize the perioperative care pathway through a combination of strategies designed to minimize surgical stress, enhance recovery, and improve postoperative outcomes. Components of ERAS that are particularly effective in preventing delirium include minimizing opioid use, promoting regional anesthesia, and ensuring early mobilization [28]. 

Regional anesthesia, such as nerve blocks, reduces the need for general anesthesia and postoperative sedatives, which are commonly implicated in delirium [29]. Early nutritional support and optimal fluid management, core tenets of ERAS, help maintain physiologic homeostasis and reduce postoperative complications. Implementing ERAS protocols results in reduced inflammatory responses and improved cognitive outcomes, directly contributing to a decreased risk of delirium [28].

Sleep disturbances are common in the postoperative period and represent a modifiable risk factor for the development of delirium. Patients often experience poor sleep quality due to pain, hospital environment factors, and interruptions from clinical staff. Disrupted sleep is associated with cognitive dysfunction, agitation, and an increased risk of delirium. Addressing sleep disturbances preoperatively and postoperatively is a key preventive measure [30]. Preoperative interventions should focus on educating patients about sleep hygiene and minimizing preoperative anxiety. Postoperatively, efforts should be made to optimize the hospital environment by ensuring quiet, dark conditions during nighttime, minimizing staff interruptions, and allowing for rest periods. Sedative use should be restricted to the minimum necessary, with alternatives such as melatonin being considered for patients experiencing significant sleep disturbances. By improving sleep quality, the risk of delirium is significantly reduced, leading to better cognitive outcomes and overall recovery [30].

In addition to individual studies, several professional bodies have issued consensus guidelines that support multimodal strategies for the prevention and management of POD. The American Geriatrics Society (AGS) recommends non-pharmacological interventions as first-line strategies, including orientation protocols, sleep hygiene, and early mobilization, consistent with the Hospital Elder Life Program (HELP). Similarly, the National Institute for Health and Care Excellence (NICE) guidelines emphasize risk assessment on admission and advocate for proactive environmental and cognitive interventions in high-risk patients. Both organizations caution against the routine use of antipsychotics, reinforcing the need for individualized, conservative pharmacologic use only when non-pharmacologic measures fail or symptoms pose safety risks.

Pharmacologic interventions for POD have produced mixed results across varying levels of evidence quality. High-quality randomized controlled trials have shown modest benefits for melatonin and its analogs in reducing delirium incidence, particularly in elderly and ICU patients. For instance, a meta-analysis of RCTs found that melatonin reduced delirium incidence with a relative risk reduction of approximately 0.6. In contrast, the evidence for antipsychotics remains inconsistent: while some retrospective studies suggest reduced delirium duration with haloperidol or atypical agents, multiple RCTs have failed to show a preventive benefit. Dexmedetomidine, a selective α2-adrenergic agonist, has demonstrated efficacy in ICU-based RCTs, particularly in reducing delirium duration and facilitating extubation. However, these trials often involved small sample sizes or specific populations, limiting generalizability. Overall, it is important to distinguish the stronger evidence from RCTs versus observational findings, which may be affected by selection bias and unmeasured confounding.

Early mobilization has long been recognized as a critical component of postoperative care, with substantial benefits in preventing both physical and cognitive complications, including delirium. Bed rest, though historically recommended after surgery, is associated with physical deconditioning and worsened cognitive function. Encouraging patients to engage in early physical activity as soon as they are medically stable is a key strategy in preventing delirium.

Mobilization helps enhance blood circulation, stimulate the central nervous system, and improve mood, all of which are protective against delirium. It also supports the restoration of regular sleep-wake cycles and mitigates the effects of prolonged bed rest. Gradual activity, starting with sitting up in bed and progressing to walking, helps maintain functional independence and reduces the incidence of postoperative complications, including delirium [31].

Disruptions in fluid and electrolyte balance are well-established contributors to delirium, particularly in older adults. Dehydration, hyponatremia, and hypernatremia can precipitate or exacerbate cognitive dysfunction. Additionally, deficiencies in thiamine and other B vitamins, especially in malnourished or alcohol-dependent individuals, can lead to Wernicke encephalopathy or other neurocognitive complications. Perioperative protocols should include careful fluid management and consideration of vitamin repletion, particularly in high-risk populations.

Family involvement in the perioperative care process is critical for preventing and managing POD. Family members can provide essential baseline cognitive information, which helps in the early identification of delirium symptoms. Their presence provides emotional reassurance and familiarizes the patient with their surroundings, mitigating confusion and anxiety [32]. In addition, family members can assist in monitoring the patient’s cognitive status and ensuring adherence to rehabilitation protocols, including early mobilization and medication management. Educating families about the signs and symptoms of delirium allows them to act as active participants in care, further reducing the risk of POD. Their involvement has been demonstrated to improve patient outcomes, decrease the incidence of delirium, and expedite recovery.

The prevention of POD requires a multifactorial approach that incorporates preoperative risk assessment, medication optimization, ERAS protocols, sleep management, early mobilization, and family involvement. A multidisciplinary team approach, with coordinated efforts across these strategies, has been shown to reduce the risk of delirium and improve postoperative outcomes significantly. As our understanding of delirium and its risk factors continues to evolve, these evidence-based strategies will remain essential in enhancing patient care and minimizing the burden of POCD [33].

Several large-scale studies have demonstrated the effectiveness of multicomponent non-pharmacological strategies in preventing POD. The Hospital Elder Life Program (HELP), in particular, has shown success in reducing delirium incidence by targeting risk factors such as sleep deprivation, immobility, sensory impairment, and dehydration. In a cluster-randomized trial by Inouye et al., the HELP intervention led to a 40% reduction in delirium incidence among hospitalized older adults. However, widespread implementation of such programs faces practical challenges, including the need for sustained staffing, training of personnel, and institutional buy-in. Adherence to protocols may also vary across healthcare settings, limiting scalability. These barriers highlight the importance of institutional commitment and resource allocation to achieve long-term impact.

In addition to incidence and resolution, POD has significant long-term consequences for patients. Multiple studies have linked POD with persistent cognitive decline, diminished functional independence, and reduced quality of life, particularly among older adults. For example, Saczynski et al. found that patients who experienced delirium postoperatively had a significantly higher risk of long-term cognitive impairment compared to those who did not. Functional outcomes such as loss of mobility, increased dependence in activities of daily living (ADLs), and institutionalization are also more common in POD patients. These findings underscore the importance of preventing and mitigating delirium not only to address short-term complications but also to preserve long-term patient-centered outcomes.

Management and treatment

Managing POD requires a multifaceted approach that incorporates both nonpharmacological and pharmacological interventions. Early detection and treatment are essential for improving patient outcomes [34].

Nonpharmacological strategies are the first-line prophylactic treatment for dealing with POD, aiming to minimize triggers and create an environment conducive to recovery. Methods such as ensuring adequate lighting by opening blinds and letting the sun in during the day or minimizing noise at night by wearing earplugs help maintain circadian rhythms and support sleep. Personalizing the space with familiar items, such as photographs, and allowing family presence can also enhance orientation and comfort. Frequent reorientation through clear, simple communication can help alleviate confusion. Family involvement is key, as it provides cognitive support and can assist in reorienting the patient to time, place, and identity [35]. Physical inactivity contributes to the development of delirium, as it exacerbates deconditioning and impairs cognitive function. Early mobilization, once the patient is stable, can help prevent POD. Simple interventions, such as sitting up, standing, and gradual walking, promote circulation and reduce sedative use, all of which support cognitive recovery [36]. Disrupted sleep is also a significant risk factor for POD. To support optimal sleep, minimizing light and noise at night, limiting unnecessary nighttime interventions, and maintaining a consistent sleep-wake cycle are essential [37].

While nonpharmacological interventions are the foundation of POD management, pharmacological treatment may be necessary in patients with severe agitation or distress. Medications are used to manage symptoms and prevent harm [34]. Haloperidol, a typical antipsychotic, is commonly used to manage severe agitation, aggression, and psychosis associated with delirium. It works as a dopamine antagonist, effectively controlling acute symptoms. However, haloperidol should be used with caution, particularly in older adults, due to the risk of exacerbating cognitive impairment and causing extrapyramidal side effects such as tremors. Its use should be reserved for patients whose agitation cannot be managed with nonpharmacological interventions [38]. Dexmedetomidine, an alpha-2 adrenergic agonist, is increasingly used as a sedative in the ICU and postoperative settings. Unlike benzodiazepines and other sedatives, dexmedetomidine does not cause respiratory depression or significantly impair cognition. It offers a sedative effect while allowing for patient interaction, which is beneficial for early reorientation. This makes it particularly useful for mechanically ventilated patients or those at high risk of delirium [39]. 

Managing POD in the ICU setting is complex due to the severity of the illness, the need for sedation, and the use of invasive interventions. Specialized protocols are necessary to optimize care. Early identification of delirium is crucial. Tools like the CAM for the ICU (CAM-ICU) are essential for routine screening [40]. Assessments help identify delirium early, enabling timely intervention and reducing its duration and severity [41]. Over-sedation is a well-known risk factor for delirium, particularly in the ICU. Sedation protocols should prioritize light sedation and minimize the use of benzodiazepines. Daily sedation assessments should be conducted to adjust sedation levels appropriately. Short-acting sedatives, such as dexmedetomidine, are preferred due to their minimal impact on cognition and favorable delirium outcomes [29]. Early mobilization in the ICU, when possible, is critical for preventing POD. Encouraging physical therapy teams to initiate movement as soon as feasible can reduce physical deconditioning and cognitive decline. Family involvement is equally important in ICU delirium management. Studies have shown that patients with family members at their bedside have a lower incidence of delirium. Families can help with reorientation, provide emotional support, and advocate for the patient’s needs [42].

Effective management of POD requires a comprehensive, individualized approach. Nonpharmacological interventions, such as environmental modifications, cognitive stimulation, early mobilization, and sleep hygiene, should be prioritized in the prevention and treatment of POD. Pharmacological treatments, such as haloperidol and dexmedetomidine, are important tools for controlling symptoms in more severe cases but should be used cautiously to avoid further cognitive impairment. In the ICU setting, structured delirium protocols that include routine screening (CAM-ICU), sedation management (light sedation, minimize benzodiazepines), and early mobilization (physical therapy) are essential. By adopting a multifaceted, evidence-based approach, healthcare providers can significantly reduce the incidence and impact of POD, thereby improving patient outcomes and recovery [34]. See Table 1 below.

**Table 1:** Management and treatment.

Treatment approach	Description	Benefits	Risks/limitations	Typical use case / notes
Nonpharmacological interventions				
Environmental optimization	Adequate lighting, noise control, familiar objects, and family presence	Maintains circadian rhythm, reduces confusion	May be insufficient alone for severe POD	The foundation of POD management
Frequent reorientation	Simple communication, family-assisted cognitive support	Reduces disorientation	Requires caregiver involvement	Useful early-stage POD
Early mobilization	Sitting up, standing, walking when stable	Reduces deconditioning, improves cognition	Patient stability must be ensured	Preventative and recovery support
Sleep hygiene	Minimize light/noise at night, consistent sleep cycle, melatonin (cautious use)	Improves sleep, reduces POD risk	Melatonin caution in the elderly	Important for prevention
Pharmacological treatments				
Haloperidol	Typical antipsychotic, dopamine antagonist	Controls severe agitation and psychosis	Extrapyramidal symptoms, cognitive impairment risk	Reserved for severe agitation, use cautiously in the elderly
Dexmedetomidine	Alpha-2 adrenergic agonist sedative	Sedates without respiratory depression, allows interaction	Cost, need for monitoring	Preferred ICU sedative, especially for ventilated patients
ICU-specific protocols				
Routine delirium screening (CAM-ICU)	Regular assessment tools for early detection	Enables timely intervention	Requires trained staff	Standard of care in the ICU
Sedation protocols	Light sedation, minimize benzodiazepines	Reduces delirium risk, maintains cognition	Potential under-sedation risk	Tailored sedation, daily assessment recommended
Early ICU mobilization	Physical therapy and movement as early as possible	Reduces deconditioning, improves cognition	Patient stability essential	Crucial in critically ill patients
Family involvement	Family presence for cognitive and emotional support	Lowers delirium incidence	May not always be feasible	Important supportive role

Outcomes and long-term impact

POD exerts consequences that extend well beyond the immediate surgical admission, reshaping recovery trajectories for patients, caregivers, and health‑care systems. Large prospective cohorts demonstrate that older adults who develop delirium remain in the hospital for three to five days longer than matched controls, even after adjustment for case‑mix and complications [1]. A prolonged length of stay exposes patients to additional hazards, including immobility, deconditioning, and nosocomial infections, and drives higher direct costs. Nationwide, excess annual expenditures attributable to delirium exceed USD 38 billion [11].

The neurologic impact is equally profound. In landmark longitudinal studies of cardiac and orthopedic patients, a single episode of delirium doubles the odds of incident dementia at one year and accelerates global cognitive decline for up to five years [43,44]. Mechanistic work implicates microglial activation, sustained neuroinflammation, and blood-brain‑barrier dysfunction as biological drivers of this persistent injury [45]. Functional ramifications quickly follow: rates of discharge to skilled‑nursing facilities rise from 11% in nondelirious patients to nearly 40% after POD, and fewer than one-third ever regain baseline activities of daily living [46]. Repercussions are not limited to patients. Qualitative studies reveal that family caregivers experience heightened psychological distress, guilt, and financial strain when delirium delays rehabilitation or necessitates institutionalization [47]. Caregiver burden, in turn, predicts poorer adherence to secondary‑prevention measures and an elevated risk of unplanned readmission [48].

At a population level, POD is an independent predictor of mortality. A meta-analysis encompassing more than 40,000 surgical patients reported hazard ratios of 1.95 for 12-month death and 1.36 for three-year death after controlling for age, comorbidity, and operative factors [49]. Proposed mediators include persistent systemic inflammation, cardiac autonomic dysregulation, and accelerated frailty progression. Taken together, these data confirm that preventing delirium is not merely a matter of reducing transient confusion; it represents a critical target for improving long-term brain health, functional independence, and survival. Interventions that demonstrably lower delirium incidence, multicomponent non‑pharmacologic bundles, depth‑of‑anesthesia titration, and perioperative dexmedetomidine, therefore, hold promise for durable outcome gains long after the patient leaves the operating theatre. 

## Conclusions

POD remains a stubborn but largely modifiable driver of perioperative morbidity and hospital resource utilization. Its pathogenesis is multifactorial, intertwining surgical stress, neuro‑inflammation, disrupted sleep-wake cycles, and pre‑existing patient frailty, yet each contributing pathway presents an opportunity for intervention. Equally compelling is the emerging role of intraoperative electroencephalographic depth‑of‑anesthesia monitoring; avoiding excessive burst suppression appears to protect vulnerable brains. Multicomponent, nurse‑led postoperative bundles that prioritize early mobilization, cognitive orientation, and environmental optimization consistently deliver clinically meaningful benefits, reinforcing that delirium prevention is truly a team sport.

Importantly, the downstream impact of POD extends well beyond the hospital stay. Patients who experience delirium are more likely to suffer persistent functional decline, accelerated cognitive trajectory toward dementia, and increased institutionalization rates. The toll on caregivers, both emotional and financial, is substantial, underscoring the societal cost of failing to address POD as a public health priority. Hospitals that have implemented systematic delirium programs report shorter intensive care and overall lengths of stay, reduced readmissions, and lower long‑term mortality, illustrating that quality improvement aligns with economic stewardship. Looking forward, real‑time risk prediction models that integrate electronic health record data with physiologic monitoring could allow truly personalized preventive strategies. Likewise, evolving insights into neuroinflammatory cascades may yield targeted pharmacologic adjuncts that complement non‑pharmacologic core measures. High‑priority research directions include large pragmatic trials to validate scalable prevention bundles across diverse surgical populations and long‑term studies that track cognitive and functional outcomes years after the index procedure.

In summary, POD highlights the importance of translating advances in neuroscience into practical perioperative care. Through careful risk assessment and the application of evidence-based prevention and treatment strategies, clinicians can meaningfully lessen its impact on patients, families, and healthcare systems. Best practices such as patient reorientation with clocks, calendars, and family involvement, maintaining uninterrupted nighttime sleep, promoting natural light during the day, and encouraging early mobilization remain central to reducing delirium in the postoperative setting. Framing POD as a critical quality metric rather than an unavoidable complication will help ensure that surgical progress is matched by improvements in long-term cognitive and functional outcomes.
